# A pilot study on the impact of known drug-drug interactions in cancer patients

**DOI:** 10.1186/s13046-015-0201-2

**Published:** 2015-08-25

**Authors:** Silvia Ussai, Riccardo Petelin, Antonio Giordano, Mario Malinconico, Donatella Cirillo, Francesca Pentimalli

**Affiliations:** Young Against Pain (YAP) Group, Parma, Italy; Sbarro Institute for Cancer Research and Molecular Medicine, Center for Biotechnology, College of Science and Technology, Temple University, Philadelphia, PA USA; Medigenia, Distretto BioHighTech FVG, Gorizia, Italy; Department of Medicine, Surgery and Neuroscience, University of Siena and Istituto Toscano Tumori (ITT), Siena, Italy; Oncology Research Center of Mercogliano (CROM), Istituto Nazionale Tumori ‘‘Fodazione G. Pascale’’ - IRCCS, Naples, Italy

**Keywords:** Drug-drug interaction (DDI), Drug toxicity, Clinical relevance, Adverse drug reactions, Oncology

## Abstract

**Background:**

When a patient concomitantly uses two or more drugs, a drug-drug interaction (DDI) can possibly occur, potentially leading to an increased or decreased clinical effect of a given treatment. Cancer patients are at high risk of such interactions because they commonly receive multiple medications. Moreover, most cancer patients are elderly and require additional medications for comorbidities. Aim of this preliminary observational study was to evaluate the incidence of well known and established DDIs in a cohort of cancer outpatients undergoing multiple treatments.

**Methods:**

Anamnestic and clinical data were collected for 64 adult patients in the ambulatory setting with malignant solid tumors who were receiving systemic anticancer treatment.

Patients also declared all drugs prescribed by other specialists or self-taken in the previous 2 weeks. DDIs were divided into two different groups: ‘neoplastic DDIs’ (NDDIs), involving antitumoral drugs, and ‘not neoplastic DDIs’ (nDDIs), involving all other classes of drugs. The severity of DDIs was classified as major, moderate and minor, according to the ‘Institute for Pharmacological Research Mario Negri’ definition.

**Results:**

About 34 % of cancer outpatients within our cohort were prescribed/assumed interacting drug combinations. The most frequent major NDDIs involved the anticoagulant warfarin (33 % of total NDDIs) that, in association with tamoxifen, or capecitabine and paclitaxel, increased the risk of haemorrhage. About 60 % of nDDIs involved acetylsalicylic acid.

**Conclusions:**

Overall, 16 % of DDIs were related to an A-level strength of recommendation to be avoided. The lack of effective communication among specialists and patients might have a role in determining therapeutic errors. Our pilot study, although limited by a small cohort size, highlights the urgent need of implementing the clinical management of cancer outpatients with new strategies to prevent or minimize potential harmful DDIs.

## Introduction

A drug-drug interaction (DDI) can be defined as the pharmacological or clinical event owed to the co-exposure of a drug with another drug or substance that modifies the patient’s response to therapy [1, 2]. DDIs, which result from a variety of processes including pharmaceuticals, pharmacokinetic or pharmacodynamic mechanisms [3], can have different outcomes by increasing or decreasing the therapeutic efficacy, inducing adverse responses, or resulting in a unique response that does not occur when either agent is given alone [4]. The clinical consequences of DDIs depend on multiple factors, including the health status of a patient (age, comorbidity, hepatic/renal failure), the narrow therapeutic index (NTI) of drugs involved (the smaller is NTI the higher is the risk), genetic polymorphisms underlying the individual variability that influences the response to a given treatment [5–7]. Moreover, also the interactions between drugs and over-the-counter (OTC) or alternative medicines and herbs should not be underestimated [8, 9].

Pharmacovigilance (PV) is the set of methods and activities designed to detect, assess and prevent adverse effects of drugs on the market (including DDIs) or other human health problems resulting from their use. In Italy, PV is required by law since 2003 (D. Leg. 95/8 April 2003) and engages healthcare professionals to promptly report adverse reactions caused by drugs. PV is therefore essentially an ex-post service: it detects drugs side effects once they appeared and keeps track of them. This traditional approach has two main limitations: first, the reports are late, since by definition monitoring consists in observing a reaction that already occurred; second, monitoring is spontaneous, part of Good Clinical Practices, and this requests a specific knowledge of the topic. As a direct consequence, underreporting is a common phenomenon in all EU countries. Even in important research centers the ratio of adverse reactions reported may not exceed 10 % (www.farmacovigilanza.org). Delays in the identification and underreporting of adverse reactions lead to a poor estimate of the magnitude of the problem, which can potentially have severe consequences on human health.

Cancer patients are at high risk to be exposed to potentially harmful DDIs because they commonly receive multiple medications, including cytotoxic chemotherapy, hormonal agents and supportive care drugs. In addition, as cancer is often associated with ageing, the age-related decline in hepatic and renal function reduces patient ability to metabolize and clear drugs, therefore increasing the potential for drug toxicity [10–12].

Moreover, the prevention of errors in the pharmacological management of cancer patients is often hampered by an ineffective communication between specialists and/or patients [13, 14]. A cancer patient may not report to the oncologist a change occurred in his/her hypertension therapy or the use of complementary/alternative medicines [15]. This ‘open loop’ management can potentially cause a late detection of DDIs and have detrimental effects. [16–18].

The evidence available to guide practitioners in the decision-making process is complex and consists of a range of sources including adverse event database entries, spontaneous or case reports, in vivo and in vitro drug metabolism studies, and in vivo drug interaction studies in healthy subjects and patients. In the absence of further rigorous studies to assess the clinical significance of DDIs, an evidence-based appraisal of the current literature is essential to guide practitioners involved in patient care. So, the main aim of our study was to assess the evidence-based level of the DDIs detected based on the current literature and evaluate their prevalence in a cohort of cancer outpatients..

## Materials and methods

### Patients features

Eligible patients were 64 adults in the ambulatory setting with malignant solid tumors treated with systemic anticancer therapies from Jan 2013 to June 2013. All patients recruited in the study had a performance status (ECOG) less than or equal to two. Our inclusion criteria excluded patients involved in any other clinical trial.

Anamnestic and clinical data were collected on age, sex, diagnosis and cancer treatment, comorbidity. Patients also declared any medication prescribed or self-taken in the 2 weeks previous to study enrollment (Table 1).

**Table 1:** Population study

Number of patients: 64
Setting: day care ambulatory
Performance status (ecog): ≤ 2
Median age: 68 years
Sex:
- Men 31 %
- Women 69 %
Type of tumors:
- Breast cancer 43 %
- Colorectal tumors 27 %
- Head and neck tumors 18 %
- Others 12 %
Concomitant disease: (average number 2)
- Cardiovascular disease 41 %
- Diabetes 26 %
- Depression 13 %
Number of medications: (average number 7)
- 2 metabolized by p-450 cyp

### Drug-drug interaction ranking and assessment

DDIs were divided into two different groups: neoplastic DDI (NDDIs), involving at least one antitumoral drug, and not neoplastic DDI (nDDIs), involving all other classes of drugs.

DDIs were ranked by pharmacological mechanisms and levels of severity [13, 19].

According to the ‘Institute for Pharmacological Research Mario Negri’ definition, severity of DDIs was classified in major, moderate and minimum, as follows:

- *minimum,* requiring no suspension or change in therapy;
- *moderate*, requiring medical attention;
- *major*, resulting in clinical consequences such as death risk and hospitalization (owing to strong hypotension, electrolytic exchange, incoercible nausea and belch, strong dehydration).

An objective of our study was to describe the evidence-based assessment of the DDIs recorded. Using a standard information set for each DDI (eg, from product labeling, textbooks, and the medical literature), a multidisciplinary group (physicians, pharmacists, biologists) assessed whether the individual drug-drug combination had an A-level strength of recommendation to be avoided.

Drug combinations were analyzed for possible chemical and physical incompatibility but these were excluded.

The study, performed in accordance with the Declaration of Helsinki has been approved by the internal ethical committee presided by Dr Giuseppe Giagnorio (prot. #01/2013).

## Results

### Patient features

The study enrolled 64 cancer patients with a mean age of 68 years, 69 % women and 31 % men who were treated in the ambulatory setting with systemic therapies (Table 1). The most common tumors were breast (43 %), colo-rectal (27 %) and head and neck cancer (18 %) (Table 1). None of our patients had metastatic tumors. Tumor features are listed in Table 2.

**Table 2:** Tumor features

Breast cancer 43 %
Invasive ductal carcinoma (IDC): Stage II (84 %) Stage III (12 %)
Profiles: Luminal A (48.8 %), Luminal B/HER2- (8.7 %), Luminal B/HER2+ (17.4 %), HER2+/ER- (16.0 %) and Triple Negative (7.1 %);
Colorectal tumors 27 %:
Adenocarcinomas, Stage IIa (25 %) Stage IIb (75 %)
Head and neck tumors 18 %
Larynx (Squamous), Stage I (21 %) Stage II (79 %)
Pharynx (HPV+), Stage I (93 %) Stage II (7 %)
Others 12 %
Bladder (Stage I)
Prostate (Gleason score 7)

### Drug usage and drug-drug interaction assessment

Regarding the antitumoral treatment, 61 % of patients received i.v. chemotherapy, 29 % hormonal therapy, 10 % oral chemotherapy. Patients also declared any medication prescribed or self-taken in the 2 weeks previous to study enrollment, including OTC medications.

Our study showed that patients received an average of seven treatments and were administered an average of two drugs, which are metabolized by the same hepatic cytochrome P-450 (CYP) enzyme isoforms (Table 1). Overall, about 34 % of the patients within our cohort were prescribed/assumed interacting drug combinations.

The most common NDDIs involved the anticoagulant warfarin (33 % of total NDDIs) and the antiepileptic drug phenytoin (17 % of total NDDIs) (Table 3).

**Table 3:** NDDIs between antineoplastic drugs and other medications

Interaction	N. of cases	Description	Severity
Warfarin x Capecitabine/Paclitaxel	1 (head-neck)	Decreased dosage of warfarin required owing to an increased risk of haemorrhage	Moderate
Quinolones x Cyclophosphamide	2 (breast)	Mucositis induced by anticancer agents might alter the absorption of kinolon	Minor
Ondansetron x Cisplatin^a^	1 (colorectal)	Increased dosage of cisplatin required	Moderate
Warfarin x Tamoxifene	4 (breast)	Increased risk of haemorrhage probably due to decreased metabolism of warfarin	Major
Phenytoin x Cisplatin	2 (colorectal)	Increased dosage of phenytoin required	Major
Hydrochlorothiazide x 5-FU/cyclophosphamide	1 (bladder)	hydrochlorothiazide may prolong chemotherapy induced neutropenia	Moderate
Furosemide x Cisplatin	1 (colorectal)	Ototoxicity augmentation, unknown mechanism	Minor

^a^In therapeutic schedule high/mild belching

As for the first, the co-administration of warfarin and tamoxifen was classified as a major NDDI because it resulted in an increased risk of haemorrhage [20, 21] (Table 3). Similarly, the association of warfarin with two common antineoplastic drugs, capecitabine [22] and paclitaxel, resulted in a moderate NDDI, with patients requiring a decreased dosage of warfarin owing to an increased risk of haemorrhage (Table 3).

For as concerns phenytoin, which can be used as anticonvulsant for patients with brain cancer or metastases, its association with cisplatin also resulted in a major NDDI. Therapeutic plans based on the concomitant use of phenytoin and cisplatin risk to fail in epilepsy control owing to a decrease in phenytoin plasma concentration [23, 24] (Table 3).

Other NDDIs involved the antibiotic quinolones and cyclophosphamide; the diuretic hydrochlorothiazide and cyclophosphamide/5-FU; the antiemetic ondansetron and cisplatin; the diuretic furosemide and cisplatin (Table 3).

Major nDDIs involved acetylsalicylic acid: the co-exposure of acetylsalicylic acid and warfarin (40 % of total nDDIs) resulted in haemorrhage risk for four patients (Table 4).

**Table 4:** nDDIs described between general and OTC medications

Interaction	N. of cases	Description	Severity
Warfarin x corticosteroids	1	Increased or decreased in anticoagulant effect of warfarin, unknown mechanism	Moderate
Proton pump inhibitors x phenytoin	2	Increased dosage of anticonvulsant required	Minor
Ondansetron x Opioids	1	Severe constipation	Moderate
Acetylsalicylic acid x Warfarin	4	Increased anticoagulant effect of warfarin	Major
Acetylsalicylic acid x ACE inhibitors/beta-blockers	2	Lowering blood pressure effect of ACE inhibitors and beta-blockers may be reduced by prostaglandin synthesis inhibition	Minor

Other identified nDDIs derived from a co-exposure between: warfarin and corticosteroids; proton pomp inhibitors and phenytoin; ondansetron and opioids, acetylsalicylic acid and ACE inhibitors/beta blockers.

By assessing the evidence-based significance of the DDIs identified within this cohort (eg from product labeling, textbooks and medical literature), we found that 16 % of these DDIs were related to an A-level strength of recommendation to be avoided. Patients involved in DDIs of major severity and those involved in A-level strength DDIs converged in a unique subgroup.

## Discussion

About 20–30 % of adverse drug reactions are supposed to be caused by DDIs [1, 2, 25]. DDIs are a particularly relevant problem for cancer patients. Anticancer therapies in fact are often based on the use of multiple agents, such as cytotoxic chemotherapy, hormonal drugs and palliative drugs used to reduce the toxicity associated with chemotherapy. These might add to the medications used to treat comorbid conditions such as cardiovascular, gastrointestinal, and other diseases. So, considering that the risk to be exposed to a DDI is approximately 50 % for patients taking five medications or nearly 100 % for patients taking seven medications [25, 26], cancer patients are at a particularly high risk to experience a DDI.

To identify DDIs in cancer patients and prevent them to occur, however, is challenging. First, there is the issue of lack of communications between specialists (oncologists and family doctors or other specialists devoted to the patient care). Second, patients could take herbs or other remedies of which their doctors are not aware but might interfere with their therapeutic protocols. Third, toxic effects owed to detrimental DDIs could be erroneously attributed to the side effects of chemotherapy and therefore underestimated. Finally, chemotherapy agents are not easily manageable because of their narrow therapeutic index, which makes hard to modulate the dosage without affecting efficacy or adverse effects.

Additionally, studies analyzing DDIs in cancer patients are hampered by several limitations inherent to the methodology involved. The major limit of the studies that screen the patient’s medications for DDIs is the shortage of information about the number of such interactions leading to adverse clinical events.

A recent study investigated the prevalence of potential DDIs among cancer patients on oral anticancer treatment [27]. Various DDIs were identified with respect to widely used antineoplastic agents such as capecitabine, lapatinib, thalidomide, and others, interacting respectively with warfarin, CYP2C8 and CYP3A4 inhibitors and benzodiazepines or other chemotherapy drugs (i.e. dexamethasone or doxorubicin) [25]. Therefore it is advisable to pay close attention to cancer patients taking these drugs and, if feasible, safer alternatives should be prescribed. In case a medication cannot be substituted, patients taking these drugs should be closely monitored for adverse events and DDIs [26, 28].

Here, we set out to evaluate the prevalence of DDIs in a cohort of 64 patients with malignant solid tumors who were treated with systemic anticancer therapies in the ambulatory setting. In particular, we focused on the interactions between anticancer drugs for which solid literature evidences exist. The patients enrolled in our study were treated for breast (43 %), colo-rectal (27 %) and head and neck cancer (18 %) and had on average two concomitant diseases (in particular cardiovascular diseases, diabetes, depression).

Our study found that the cancer patients within our cohort, who had a median age of 68 years, received an average of seven treatments and were administered an average of two drugs metabolized by the same hepatic cytochrome P-450 (CYP) enzyme isoforms, which is consistent with other studies analyzing polypharmacy in elderly cancer patients [29, 30]. We found that 34 % of these patients were prescribed/assumed interacting drug combinations.

The most common NDDIs involved the anticoagulant warfarin (33 % of total NDDIs) and the antiepileptic drug phenytoin (16 % of total NDDIs), whereas major nDDIs involved acetylsalicylic acid. In particular, the co-exposure of acetylsalicylic acid and warfarin (40 % of total nDDIs) resulted in haemorrhage risk for four patients.

Patients receiving drugs interacting with warfarin face the risk of increases in prothrombin time international normalized ratio (PT-INR) values and subsequent haemorrhage. Various studies analyzed the effect of capecitabine and warfarin combination showing that capecitabine affects the anticoagulant effect of warfarin not only during the co-administered term but also after discontinuation term, suggesting that PT INR levels should be closely monitored in patients using these drugs together [31, 32].

Similarly, we found association of warfarin with tamoxifen, which is used as hormonal treatment for women with oestrogen-receptor-positive breast cancer. Tamoxifen therapy and cancer can increase the risk of venous thromboembolism, however the concomitant use of warfarin is contraindicated, because it increases the risk of bleeding complications and requires consistent and careful monitoring of the coagulation profile [20, 33].

So, in those cases for which it might not be possible to recommend alternative medications, it should be necessary at least to monitor carefully the status of patients treated with the contraindicated drugs.

Aggressive dosing of phenytoin could be required to achieve therapeutic concentrations in patients who concurrently receive chemotherapy such as cisplatin) and/or dexamethasone, especially in patients who fall outside the predictive pharmacokinetic model for phenytoin. Subtherapeutic phenytoin concentrations may be decreased by concomitant use of cisplatin owing to variousmechanisms and therefore its use needs to be closely monitored [23, 24].

A-level evidence shows that concomitant use of NSAIDs in anticoagulated carries a real risk of serious bleeding, as well as thromboembolism. Thus, physicians should clearly exercise extra caution with NSAIDs in patients with cancer, especially if they are anticoagulated. Also, cancer patients with NSAIDs should also undergo regular clinical review, and clinicians should regularly reassess the need for NSAID use. Finally, as a part of regular clinical assessment, bleeding risk should be routinely assessed, and the HAS-BLED score is now recommended in many guidelines for this purpose.

Our study showed that, based on the current evidence, 16 % of the DDIs identified in our cohort were related to an A-level (high) strength of recommendation to be avoided. It is likely that our findings could be extended to other tumor types such as lung, pancreatic and other cancers for which patients heavily depend on chemotherapy. The rates of DDIs is likely to vary depending on various factors including: *i*) tumor stage: in our cohort we did not have metastatic patients; patients with early stage cancer are less likely to take multiple medications; *ii*) adult and older age patients are more likely to have higher comorbidities than younger cancer patients. Nonetheless our findings strongly suggest that the DDI issue needs to be taken in serious consideration when dealing with cancer outpatients.

The lack of effective communication among specialists and patients might have a role in the therapeutic errors involving DDIs.

## Conclusions

Overall, by showing that 34 % of cancer patients were prescribed/assumed interacting drug combinations of which 16 % were recommended to be avoided, our data suggest that, not only it is important to improve our knowledge regarding the effects derived from a co-exposure to drugs, but it is very urgent to implement the clinical management of patients based on the current evidence of well-established and potentially harmful DDIs. This could be achieved by improving the communication among all the doctors taking care of a patient or establishing a ‘connectivity unit’. Some studies in particular propose that the role of clinical pharmacists, possibly with a specific training in oncology [34], could be implemented to reduce medication related problems and optimize therapeutic treatments [35]. Alternatively, it should be considered the opportunity to generate new fully-automated methods, through the use of modern information technology, to control the pharmacological risk that are independent from medical communication, in order to decrease the number of toxicological drug-related consequences. An example in this direction is the development of specific databases to detect DDIs in primary healthcare [36]. Furthermore, all the stakeholders should commit to sensitize patients, through ad hoc health education campaigns, in order to reduce potential causes of interference with medications for chemotherapy or comorbidities, such as OTC, herbs or other remedies.

Ultimately, it will be important to investigate more thoroughly the real impact of DDIs in oncology, by assessing how the common DDIs involving genetic modifications can occur and also the economic impact of DDIs on the health system. For these purposes large representative epidemiologic studies are needed [37].
